# Antidepressant discontinuation manias: a new bipolar subtype?

**DOI:** 10.1192/j.eurpsy.2022.309

**Published:** 2022-09-01

**Authors:** S. Abou Kassm, W. Naja

**Affiliations:** 1 Center Hospitalier Guillaume Regnier, Brittany, Rennes, France; 2 King Hussein Cancer Center, King Hussein Cancer Center, Amman, Jordan

**Keywords:** antidepressant, discontinuation, bipolar III 1/4, mania

## Abstract

**Introduction:**

Antidepressant withdrawal manic states are rare and controversial phenomena. The underlying pathophysiology and the clinical implications have not been thoroughly discussed in the literature.

**Objectives:**

We aimed to review reports of antidepressant discontinuation manic states and to discuss the different hypothetical pathophysiological changes underlying this phenomenon. We also argued in favor of its inclusion in the bipolar spectrum.

**Methods:**

We searched Pubmed using the key words: ‘antidepressant withdrawal’ or ‘antidepressant discontinuation’ plus ‘mania’ or ‘hypomania’ from January 2008 until January 2018.

**Results:**

Twenty-nine cases of antidepressant discontinuation manic states were identified. Hypotheses involve the implication of Catecholamines, Acetylcholine and Serotonin in the pathophysiology of this paradoxical phenomenon. The search for red flags for bipolar disorder in these case reports revealed psychiatric histories in favor of a bipolar spectrum disorder in 12 individuals while five were already known to have bipolar disorder.

**Conclusions:**

Antidepressant discontinuation mania should be considered on the bipolar spectrum.

**Disclosure:**

No significant relationships.

